# A comparative study on 3D printing-assisted arthroscopic IDEAL point femoral tunnel positioning for anterior cruciate ligament reconstruction versus conventional arthroscopic positioning

**DOI:** 10.1186/s12891-024-07591-y

**Published:** 2024-06-19

**Authors:** Tiezhu Chen, Junjie Chen, Xiaosheng Li, Yinhao He, Qiang Peng, Hongwen Chen

**Affiliations:** 1 Department of Orthopedics, Hunan Provincial People’s Hospital, The First Affiliated Hospital of Hunan Normal University), Changsha, Hunan 410002 China; 2 Department of Orthopedics, Longhui People’s Hospital, Longhui, Hunan 422200 China; 3 Clinical Research Center of Sports Medicine in Hunan Province, Changsha, 410002 China

**Keywords:** Anterior cruciate ligament reconstruction, 3D printing, IDEAL femoral tunnel, CT-guided reconstruction

## Abstract

**Background:**

This study aimed to investigate the feasibility and precision of using a 3D-printed template for femoral tunnel placement in guiding the optimal positioning of the Internal anatomical stop and Low tension maintenance (IDEAL) bone tunnel during single-bundle anterior cruciate ligament (ACL) reconstruction.

**Methods:**

A retrospective analysis was conducted on 40 patients who underwent arthroscopic single-bundle ACL reconstruction at our hospital between April 2021 and November 2021. In the direct vision group, the IDEAL bone tunnel was positioned using radiofrequency localization directly visualized at the stump. In the 3D-printed positioning group, preoperative CT scans and Digital Imaging and Communications in Medicine (DICOM) data were employed. Following the Quadrant method by Bernard, the femoral tunnel’s depth was set at 25% and its height at 29%. Postoperative plain CT scans enabled the reconstruction of 3D models for both groups. The accuracy of femoral tunnel placement was then compared.

**Results:**

The central locations of the bone tunnels in the direct vision group were at a mean depth of 25.74 ± 1.84% and a height of 29.22 ± 2.97%. In the 3D printing localization group, these values were 25.39 ± 2.98% for depth and 28.89 ± 2.50% for height, respectively. No significant differences were found in tunnel positioning between the groups. Both groups demonstrated statistically significant improvements in International Knee Documentation Committee Subjective Knee Form (IKDC) and Lysholm scores postoperatively, with no significant differences observed 12 months post-surgery.

**Conclusion:**

The findings of this study suggest that 3D printing-assisted arthroscopic IDEAL point femoral tunnel positioning and conventional arthroscopic positioning are feasible and effective for ACL reconstruction. Using 3D printing technology to design femoral anchor points in ACL reconstruction allows for the customization of anterior fork reconstruction and precise bone tunnel positioning, supporting the goal of individualized and accurate reconstruction.

## Background

Injuries to the anterior cruciate ligament (ACL), frequently incurred during sports activities, typically lead to symptoms such as knee instability and locking [1]. These injuries may also precipitate secondary damage to cartilage and the meniscus, hastening knee degeneration [2]. The primary intervention for ACL rupture is arthroscopic ACL reconstruction [3]. As surgical techniques have evolved, anatomical single-bundle reconstructions have become the clinical norm. Concurrently, methodologies for bone tunnel localization have been progressively refined [4]. Pearle et al. defined the Internal anatomical stop and Low tension maintenance (IDEAL) femoral tunnel location incorporating anatomical, histological, isometric, biomechanical, and clinical insights into the ACL femoral insertion [5]. IDEAL represents Isometry, Direct insertion of ACL, Eccentrically located, Anatomical footprint area, and Low tension in flexion, guiding precision in tunnel placement [5].

While medical professionals widely recognize the IDEAL femoral tunnel location, accurately pinpointing this site during surgical procedures presents a considerable challenge [6]. For less experienced surgeons, postoperative evaluations often reveal discrepancies between the actual and planned positions of the bone tunnel [7]. Thus, enhancing the accuracy of femoral tunnel positioning remains a critical clinical issue requiring urgent attention.

## Materials and methods

We conducted a retrospective analysis of 40 patients diagnosed with ACL rupture who underwent arthroscopic single-bundle ACL reconstruction at our institution from April 2021 to November 2021. The study cohort included 20 patients with femoral tunnel placement under direct vision and 20 patients using a 3D printing template for tunnel positioning. Postoperative computed tomography (CT) scans were used to assess the fidelity of the femoral tunnel location to that planned, using the 3D printed templates. This study aimed to evaluate the feasibility of employing a 3D printing template to achieve the IDEAL femoral tunnel location in ACL reconstructions.

### Ethics statement

This study was reviewed and approved by the ethics committee of Hunan Provincial People’s Hospital (Approval number: 202,003). Participants were randomly assigned to study groups in a manner concealed from them at the time of selection. The surgical procedures were performed by experienced surgeons to ensure high-quality outcomes. This study adopted a single-blinded methodology to maintain the integrity of the randomization.

### Inclusion and exclusion criteria

#### Inclusion criteria

Aged between 15 and 50 years; No previous history of ACL rupture and clear indications for ACL reconstruction surgery; Consent to use autologous hamstring tendon for grafting.

#### Exclusion criteria

previous ACL re-rupture; Radiographic evidence of moderate to severe knee osteoarthritis; Open growth plates; Concurrent multi-ligament knee injuries, cartilage injuries, or other conditions requiring additional surgical interventions beyond ACL reconstruction; Previous knee surgeries.

### Participants

This retrospective study analyzed 40 patients who suffered an ACL rupture and underwent arthroscopic single-bundle ACL reconstruction between April 2021 and November 2021. The participants were divided into two groups: direct vision positioning and 3D printing positioning. The allocation of participants across the groups is detailed in Table 1.

**Table 1:** Grouping details and causes of injury

Groups	No. of cases	Gender		Age (Year)	Causes of injury	
		Male	Female		Sports	Traffic accident
3D printing	20	17	3	27.55 ± 7.84	14 cases	6 cases
Direct vision	20	17	3	29.20 ± 7.42	15 cases	5 cases
P		> 0.05		> 0.05	> 0.05	

### Surgical procedure

#### Arthroscopic examination

All procedures were conducted by the same medical team. Following intubation for general anesthesia, patients were placed in a supine position with a low-pressure tourniquet applied at the base of the thigh to minimize blood flow. Standard procedures for surgical site sterilization were followed. Anteromedial and anterolateral portals were established, through which a 30-degree arthroscope was introduced to assess the joint and confirm the ACL rupture, thereby necessitating reconstruction. Upon completion of the inspection, the arthroscope was removed, and the portals were sutured closed.

#### Graft preparation

During surgery, the tibial tubercle and the medial hamstring tendons were palpated. A longitudinal incision of 3 cm was made approximately 2.5 cm medial to the tibial tubercle. The skin and subcutaneous fascia were sequentially incised, revealing the pes anserinus (“goose’s foot”). Superior to the sartorius muscle, the tendon sheath was accessed, exposing the underlying gracilis and semitendinosus tendons. These tendons were isolated, exteriorized, and meticulously cleaned using right-angle forceps. Accessory tendons were excised under wire guidance. The tendons were detached from their tibial attachments using a tendon stripper and cleared of any residual muscle tissue. Both ends of each tendon were then sutured using ETHICON suture (#2). The graft’s diameter was established by folding it in half or into thirds, aiming for an optimal diameter between 7 mm and 9 mm, with 8 mm considered ideal. The prepared grafts were securely stored for subsequent implantation.

#### Bone tunnel preparation

##### Direct vision positioning group

**Fig. 1:**
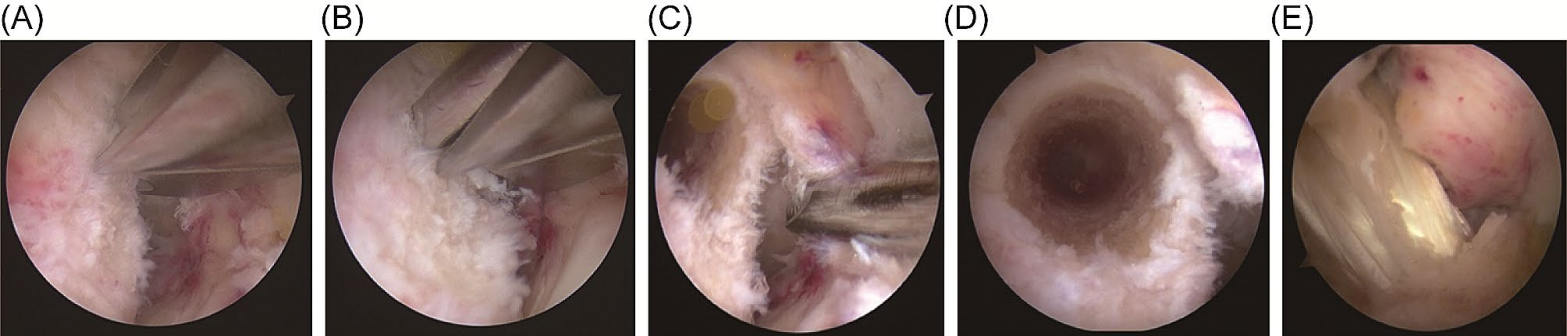
Intraoperative photographs of the direct vision positioning group. (**A**) Positioning using a traditional locator; (**B**) Drilling with a guide pin using a traditional locator; (**C**) Posterior wall of the femoral tunnel in the direct vision positioning group; (**D**) Anteroposterior view of the femoral tunnel in the direct vision positioning group; (**E**) Postoperative image following ligament reconstruction in the direct vision positioning group

The arthroscope was reinserted via the anterolateral portal, while a shaver was introduced through the anteromedial portal. Radiofrequency ablation was employed to debride the joint, enhancing the visibility of the femoral footprint. The knee was oriented in a ‘4-figure’ position. A new portal was created anteromedially and inferiorly above the medial meniscus, facilitating access to the ACL’s femoral footprint via the intercondylar notch without impinging on the femoral medial condyle. The shaver was repositioned to this new portal, and further radiofrequency ablation exposed the femoral footprint and the transition zone between the posterior cartilage wall of the femoral intercondylar notch. The IDEAL insertion point for the ACL on the femur was marked using radiofrequency and confirmed intraoperatively by two surgeons, each with over five years of experience. The arthroscope was then positioned via an anterior endotopal approach to meticulously clear the cartilage margin’s endpoint, deep synovial tissue, and ligament remnants, thereby exposing the lateral bone surface of the femoral lateral condyle. It was essential to verify that the placement of the marker met the stipulated requirements. Subsequently, the arthroscope was switched to the anterolateral approach for improved visualization. A 6 mm eccentric guide (Smith & Nephew, USA) was introduced through the anterior submedial approach, anchoring at the cartilage transition of the posterior wall of the intercondylar fossa. With the knee flexed beyond 110°, the guide was adjusted to center the 2.4 mm guide needle on the marked point. An electric drill was then used to penetrate the opposing cortical bone, followed by a 4.5 mm endobutton drill (Smith & Nephew, USA) to create the precise bone tunnel. The length of the bone tunnel was measured, and based on this length and the graft diameter, a broader bone tunnel was fashioned (Fig. 1).

##### 3D printing positioning group: design and application of bone tunnel and 3D guide plate

**Fig. 2:**
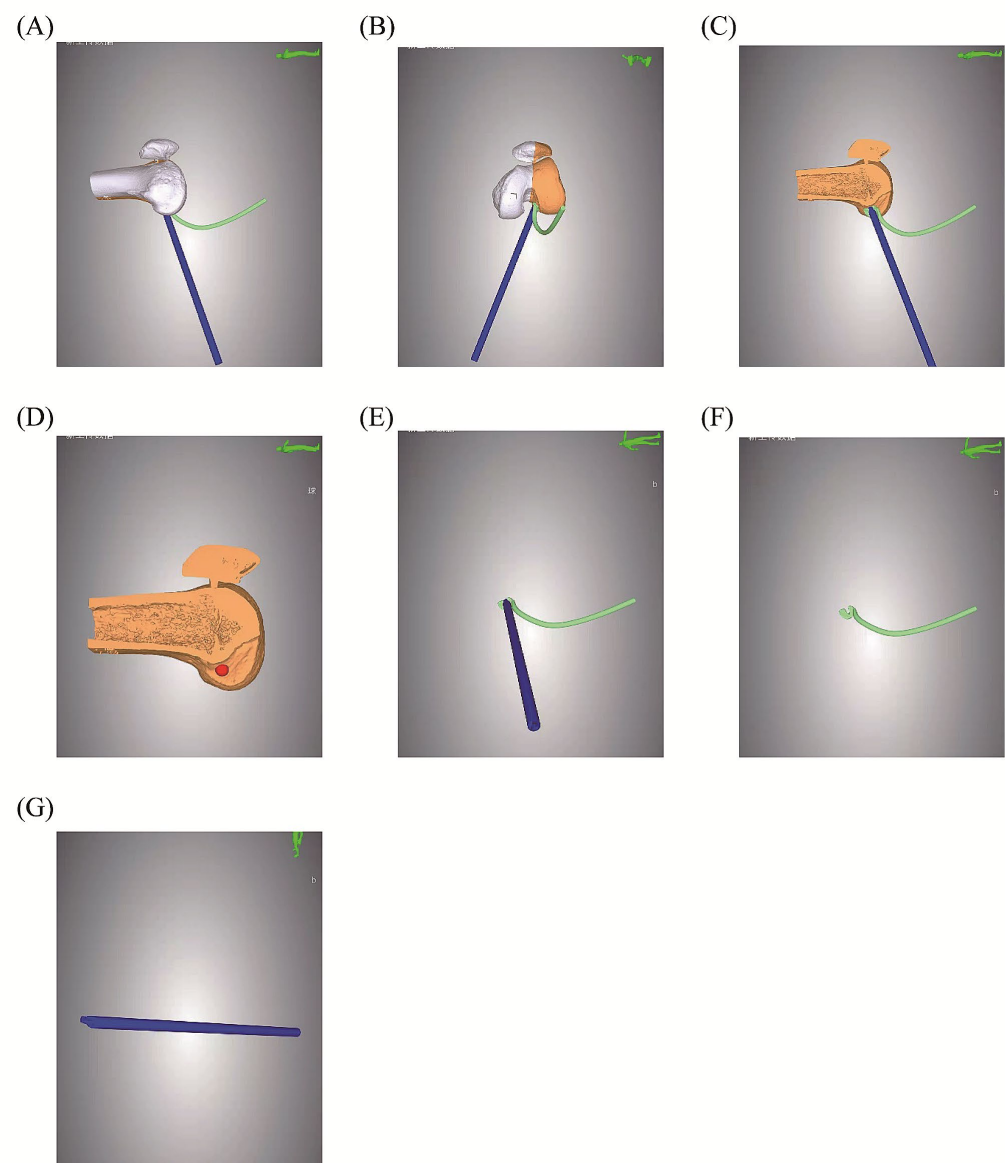
3D printing modeling. (**A**) Side view of 3D printing modeling (internal side); (**B**) 3D printing modeling of frontal image of femoral condyle; (**C**) 3D printing modeling of lateral view of medial condyle resection, revealing the attached guide plate, hollow guide and positioning site; (**D**) 3D printing modeling of lateral view of excision of medial condyle and its location site; (**E**) attach guide plate and hollow guide; (**F**) attach guide plate; (**G**) hollow guide

**Fig. 3:**
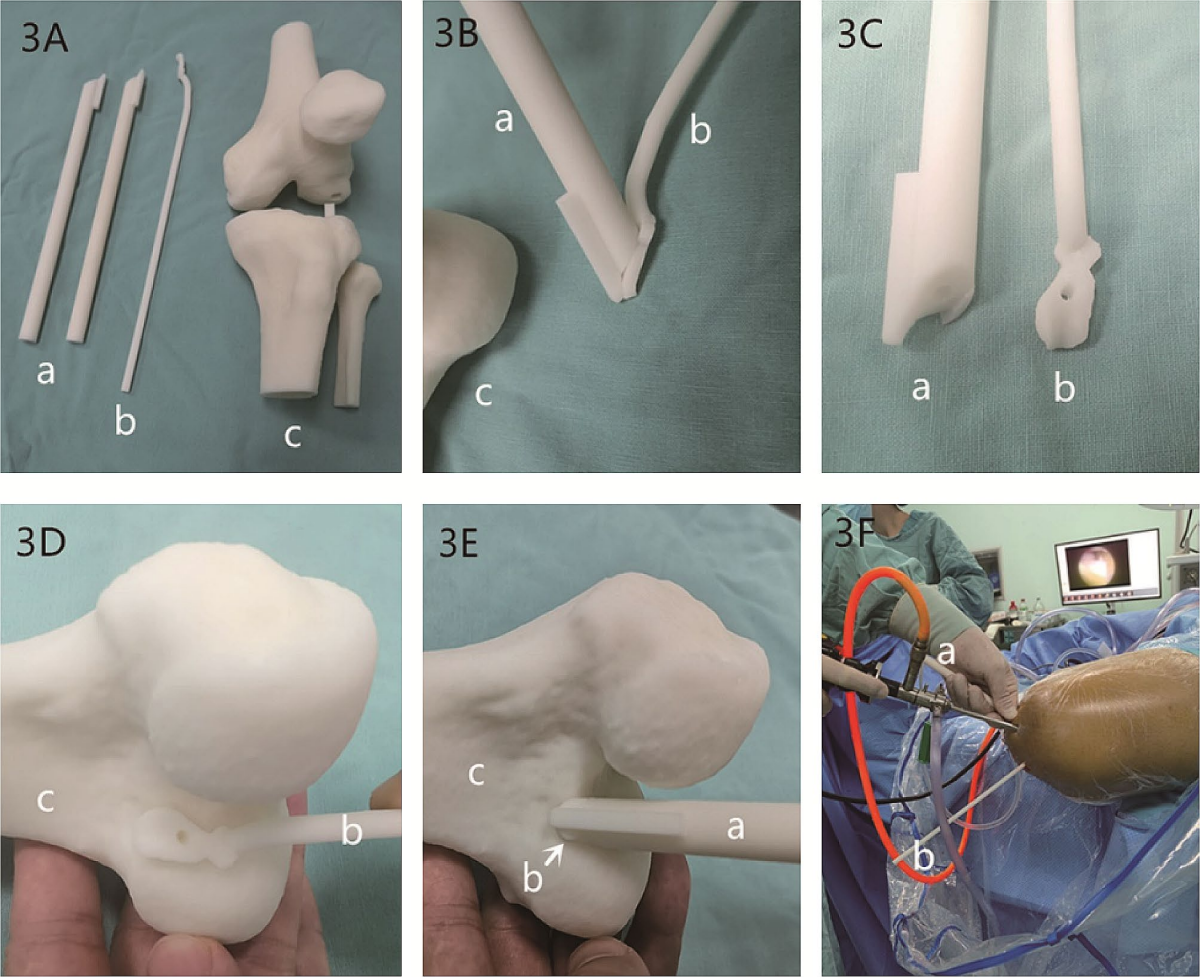
3D-printing model and images of intraoperative positioning. (**A**) 3D printing components: a, guide; b, attached guide plate; c, 3D printed model of the knee joint. (**B**) diagram showing the guidance of guide with attached guide plate: a, guide; b, attached guide plate. (**C**) cross-sectional diagram of guide a with attached guide plate: a, guide; b, attached guide plate. (**D**) 3D diagram indicating IDEAL points with attached guide plate: b, attached guide plate; c, 3D printed model of the femoral knee joint. (**E**) diagram showing the positioning of IDEAL points using guide a attached to guide plate b: a, guide; b, attached guide plate; c, 3D printed model of the femoral knee joint. (**F**) intraoperative use of guide a with attached guide plate b for positioning: a—guide; b, attached guide plate

A preoperative CT scan of the knee joint was conducted, and the 3D CT Digital Imaging and Communications in Medicine (DICOM) data were imported into Mimics Research 20.0 software to produce a 3D reconstruction of the knee joint. This model data was saved as a Standard Template Library (STL) file. Subsequently, the STL files were imported into Materialise Magics 20, generating a 3D virtual knee joint model. The orientation parameters for this model included the femur’s longitudinal axis in the coronal plane as the horizontal reference, the anterior trochlear plane as the superior reference, the distal end of the femur as the anterior reference, the femoral condyle as the inferior reference, and the transition location of the posterior margin of the femoral cartilage as the posterior reference. A custom guide plate was designed to conform precisely to the lateral bone structure of the lateral femoral condyle (Fig. 2A-C). The boundaries of the guide plate were defined as follows: the upper boundary by the terminal plane of the femoral lateral condyle’s cartilage margin, the lower boundary by the cartilage margin, the deeper boundary by the apex of the posterior cartilage margin of the lateral wall of the intercondylar notch, and the shallower boundary not extending beyond the midpoint of the lateral femoral condyle. The guide plate had an approximate depth of 10 mm and a thickness of 3 mm. Employing the Quadrant method proposed by Bernard, the placement of the femoral tunnel was determined [8], with the tunnel’s internal opening positioned at 25% of the depth and 29% of the height of the lateral femoral condyle, represented by a hollow circle with a diameter of 2.4 mm (Fig. 2D, E). The external opening was positioned sagittally on the lateral slope of the femoral condyle, and the tunnel’s total depth was set between 36 and 42 mm. In alignment with the tunnel’s trajectory, a 2.4 mm hole was created in the guide plate, accompanied by a fan-shaped incision at its upper boundary, opened at an angle of 50°. The guide plate’s handle was designed with a length of 250 mm and a diameter of 5 mm, accompanying a 20 cm long and 2.4 mm diameter hollow guide. This hollow guide’s distal end fits snugly into the fan-shaped incision while its remainder conforms to the femur’s surface. The guide plate was meticulously designed to match the irregular contour of the attached guide plate (Fig. 2). **The 3D-printing model was shown in** Fig. 3.

##### 3D printing positioning group: femoral tunnel preparation

**Fig. 4:**
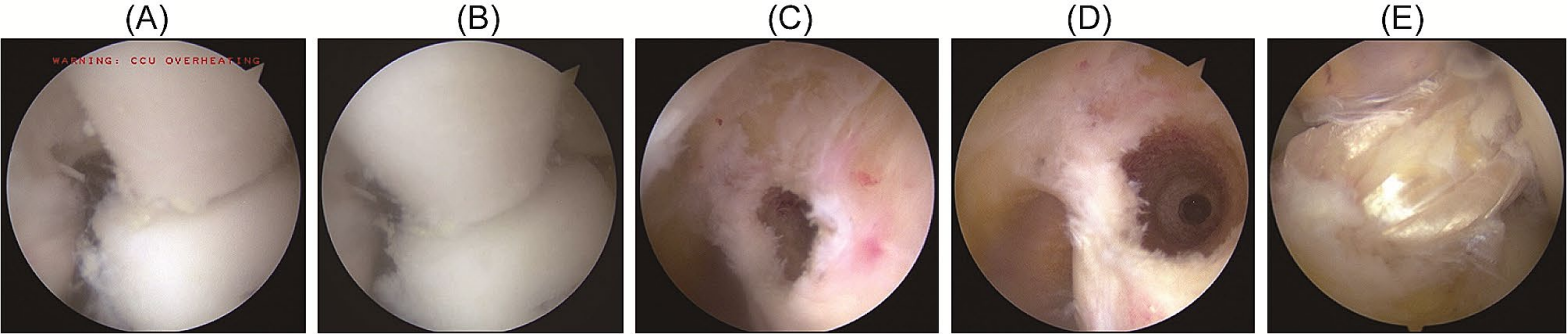
Intraoperative photographs of the 3D-printing positioning group. (**A**) Positioning using a hollow guide in the 3D-printing guide plate; (**B**) Drilling in the 3D-printing positioning group; (**C**) Posterior wall of the femoral tunnel during surgery in the 3D-printing positioning group; (**D**) Anteroposterior view of the femoral tunnel in the 3D-printing positioning group; (**E**) Postoperative image following ligament reconstruction in the 3D-printing positioning group

Utilizing the previously detailed method, anterior external, anterior superior internal, and anterior inferior internal portals were established. The knee was positioned in a quadrangular arrangement. Following this methodology, the lateral bone surface of the lateral femoral condyle, the terminal point of the deep cartilage margin, the inferior cartilage margin, and the femoral stump of the ACL were meticulously cleaned and visualized. The arthroscope was introduced via the anterior medial superior portal, revealing the terminal and inferior cartilage margins of the femoral posterior condyle. The reference point was selected from the terminal to the inferior cartilage margin along the medial-lateral femoral condyle. Guide plate B was introduced through the lateral portal, and its precise placement, considering the deep boundary, the terminal cartilage point, and the inferior cartilage margin, was confirmed. Subsequently, Guide A was inserted via the anterior inferior internal portal, and the knee was flexed beyond 110°. The procedural steps were analogous to those of the direct vision positioning Group. A 2.4 mm guide needle was centered on the marked reference point, and drilling was performed through the opposite cortical bone. A 4.5 mm endobutton drill (Smith & Nephew, USA) shaped the fine bone tunnel, and its length was gauged. The larger bone tunnel was constructed based on this measurement and the graft diameter **(**Fig. 4**)**.

#### Tibial tunnel preparation

The methodology for preparing the tibial tunnel was uniformly applied across both study groups. During the procedure, selected fibers at the tibial insertion point of the anterior cruciate ligament were preserved. An ACUFEX tibia locator (Smith & Nephew, USA) set at a 55° angle was utilized. The internal opening was centrally positioned at the stump of the anterior medial band, while the external opening was placed medial to the tibial tubercle. The locator was employed to ascertain the appropriate length of the tibial bone tunnel, ensuring it was neither too short nor excessively steep. A Kirschner wire was introduced via an electric drill to establish the tibial tunnel, conforming to the diameter of the graft tendon.

#### Graft introduction and fixation

A singularly folded ETHICON suture (#5) was threaded into a long guide needle and introduced into the femoral tunnel via a medial anterior portal. The external femoral skin was subsequently sutured. From the tibial tunnel, the suture was retrieved from the anterior medial end within the joint space to the external tibial end. The endo-button traction line and loop were then pulled from the femoral end. Before insertion into the bone tunnel, the graft tendon was thoroughly rinsed with saline. The tibial end traction line was tightened, ensuring the lateral femoral loop was correctly positioned. The anterior cruciate ligament was maintained clear of the posterior horn and the intercondylar notch throughout the flexion and extension. Finally, the graft was secured with absorbable hydroxyapatite screws.

### Postoperative rehabilitation training

Postoperatively, patients were fitted with an extended brace for 8–12 weeks. Following the procedure, they were encouraged to commence ankle pump exercises and lower limb muscle isometric contractions as early as possible. Full weight-bearing was permitted from the first-day post-surgery. Range of motion exercises commenced one week post-surgery, initially targeting 0°-90° flexion and progressing to 0°-120° by one month post-surgery. By the third month post-surgery, patients achieved a range of motion comparable to that of the unaffected knee, attaining full mobility. By the sixth month, patients were encouraged to engage in swimming, brisk walking, and jogging, contingent upon the recovery of muscle strength. From the seventh to the ninth month post-operatively, activities were intensified to include fast running and general exercises tailored according to individual recovery of muscle strength. During this period, there was a focus on strengthening exercises for the quadriceps and biceps femoris muscles. Once muscle strength exceeded 80% of that of the contralateral lower limb, patients were gradually reintegrated into their pre-injury levels of activity.

### Postoperative imaging evaluation

**Fig. 5:**
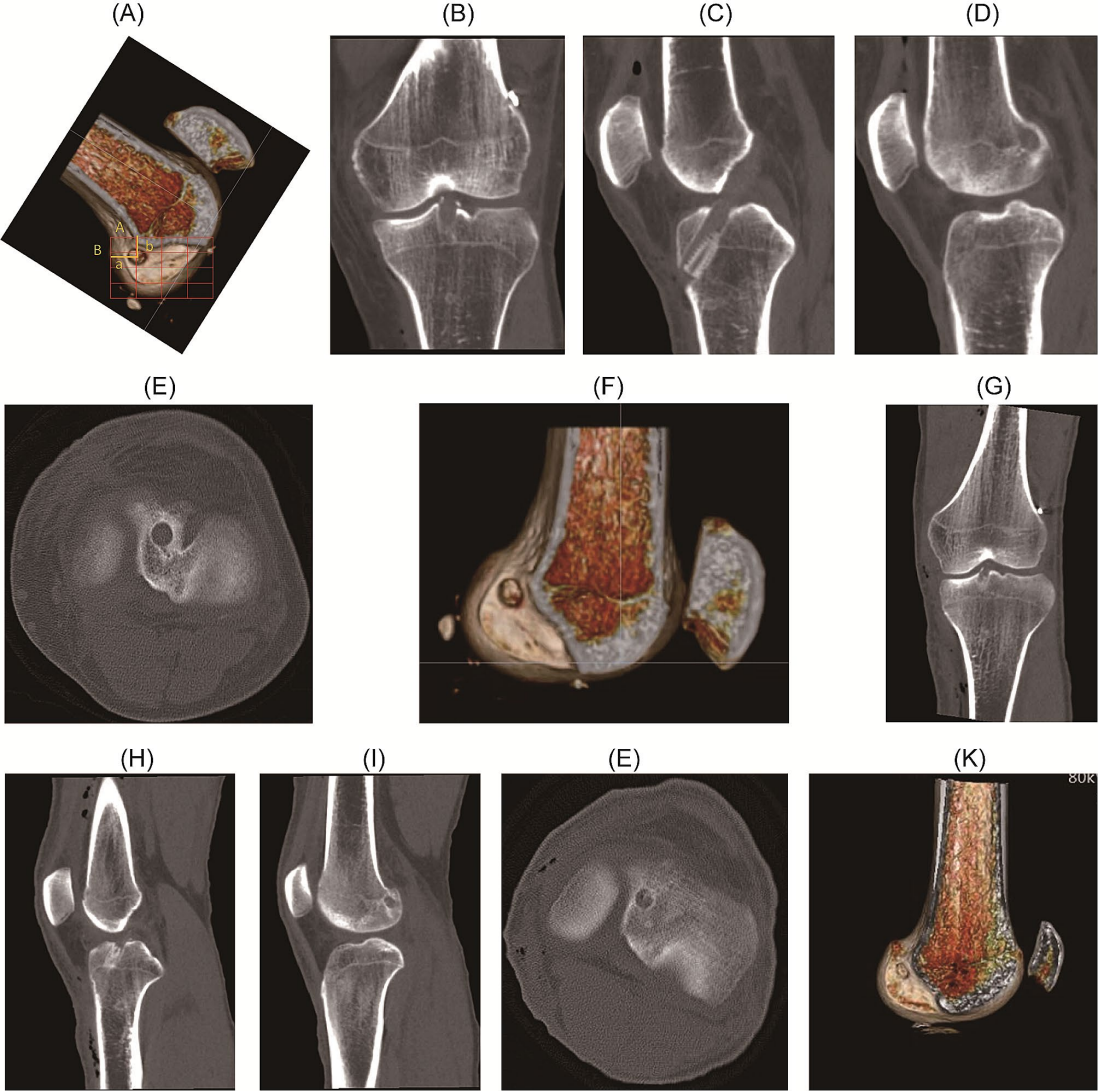
Postoperative X-ray and three-dimensional CT assessment of the tunnel position in patients of the 3D-printing and direct vision positioning groups. (**A**) Position of femoral tunnel in 3D CT (A is the tangent length of the lateral condyle of the femur, and B is the tangent height of the anterior and posterior edges of the lateral femoral condyle; a is the length of the posterior wall of the lateral femoral condyle from the center of femoral tunnel, and b is the distance between the center of the bone tunnel and the Blumensaat line). (**B**) Coronal CT scan showing the postoperative internal orifice of the tibial tunnel. (**C**) Sagittal CT scan showing the postoperative tibial tunnel. (**D**) Sagittal CT scan showing the postoperative internal orifice of the femoral tunnel at the IDEAL point. (**E**) Axial CT scan showing the postoperative internal orifice of the tibial tunnel. (**F**) 3D CT reconstruction showing the position of the internal orifice of the femoral tunnel at the IDEAL point. Postoperative CT reviews following anterior cruciate ligament reconstruction show that the femoral bone tunnel orifice is located at the IDEAL point on the medial side of the lateral femoral condyle. (**G**) Coronal CT scan showing the postoperative internal orifice of the tibial tunnel; (**H**) Sagittal CT scan showing the postoperative tibial tunnel; (**I**) Sagittal CT scan showing the postoperative internal orifice of the femoral tunnel at the IDEAL point; (**J**) Axial CT scan showing the postoperative internal orifice of the tibial tunnel; (**K**) 3D CT reconstruction showing the position of the internal orifice of the femoral tunnel at the IDEAL point. Postoperative CT reviews confirm the location of the internal orifice of the femoral bone tunnel at the IDEAL point on the medial side of the lateral femoral condyle

All patients underwent anteroposterior and lateral knee radiographs within three days following surgery. A plain CT scan facilitated the three-dimensional reconstruction of the knee joint. Measurements of femoral tunnel height and depth at the Blumensaat line were conducted. Utilizing three-dimensional CT images, specific anatomical landmarks, including the medial wall of the lateral femoral condyle, the Blumensaat line, the anterior and posterior walls of the lateral femoral condyle, and the center point of the femoral bone tunnel, were precisely identified **(**Fig. 5**)**. The Quadrant method was employed to measure the tangent length of the lateral femoral condyle (A) and the tangent height of the femoral condyle (B) **(**Fig. 5A**)**. Distances from the center of the femoral tunnel to both the posterior wall of the lateral femoral condyle (a) and the Blumensaat line (b) were quantified. The ratios of these measurements (depth a/A, height b/B) in the Quadrant were compared.

### Statistical analysis

Statistical analysis was performed using SPSS version 22.0. Measurements were presented as means ± standard deviation (SD). Data adhering to a normal distribution were compared using an independent sample t-test, while the Mann-Whitney test was applied for non-normally distributed quantitative data. Qualitative data were analyzed using a Chi-square test, with the significance level set at *P* = 0.05.

## Results

The study groups were well-matched, with no significant differences in gender or age, confirming group comparability. All 40 patients in each group were followed for 12 months. Surgical incisions healed appropriately without any complications, such as infections. Postoperative CT scans were utilized to ascertain the location of the bone tunnel, and the Quadrant method was employed to evaluate this location (Fig. 5). The central positions of the bone tunnel in the direct vision group were 25.74 ± 1.84% for depth and 29.22 ± 2.97% for height. In the 3D printing localization group, these measurements were 25.39 ± 2.98% for depth and 28.89 ± 2.50% for height. No significant statistical differences were observed between the groups (*P* > 0.05, Table 2).

**Table 2:** Comparison of femoral tunnel positioning in anterior cruciate ligament reconstruction difference between two groups (Means ± SD)

	Percentage of bone tunnel center to Line A (depth)	Percentage of bone tunnel center to Line B (height)	Full length of bone tunnel (mm)
3D printing (20 cases)	25.39 ± 2.98	28.89 ± 2.50	38.80 ± 1.77
Direct vision (20 cases)	25.74 ± 1.84	29.22 ± 2.97	39.40 ± 1.96
*t* value	-0.402	-0.370	-1.189
*P* value	0.692	0.716	0.249

Note: ACL, the anterior cruciate ligament; A, the tangent length of the lateral femoral condyle. B, the tangent height of the anterior and posterior edge of the lateral femoral condyle

**Fig. 6:**
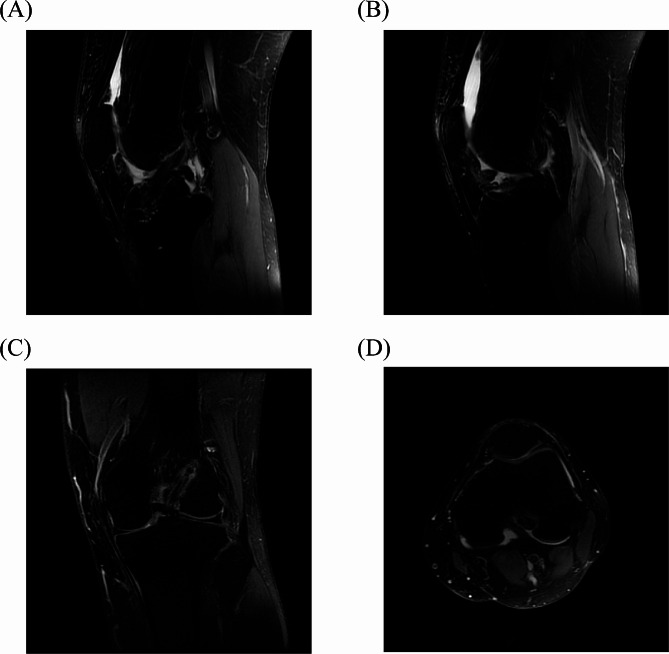
MRI images were reviewed 12 months after surgery. (**A)** Sagittal MRI showed good ACL continuity and good ligament tension after reconstruction; (**B)** Sagittal MRI showed the ligament at the femoral tunnel of ACL after reconstruction; (**C)** Coronal MRI showed the femoral intramuscular ligament of ACL after reconstruction; (**D)** MRI transverse section showed the ligament at the femoral tunnel of the ACL after reconstruction

At the 12-month follow-up, knee MRI scans were conducted to evaluate graft integrity. Both groups exhibited continuous postoperative cruciate ligaments with moderate tension; no significant kinks or ruptures were detected. The range of motion for the knee was either within or exceeded 0-120°, demonstrating satisfactory flexion and extension capabilities (Fig. 6).

An independent sample t-test was utilized to compare the IKDC and Lysholm scores across the two study groups. Within each group, the IKDC and Lysholm scores exhibited statistically significant differences when comparing pre- and post-surgery assessments (*P* < 0.05). However, no significant difference was observed in the IKDC and Lysholm scores between the two groups before and after surgery (*P* > 0.05, as detailed in Table 3).

**Table 3:** Comparison of IKDC score and Lysholm score between the two groups, before and six months after operation (Means ± SD)

	IKDC score					Lysholm score			
	Preoperative	Last follow-up	*t*	*P*		Preoperative	Last follow-up	*t*	*P*
3D printing (20 cases)	48.95 ± 6.01	88.65 ± 10.18	-20.17	0.000		47.90 ± 4.30	90.15 ± 5.23	-29.42	0.000
Direct vision (20 cases)	49.90 ± 5.52	89.40 ± 5.14	-23.36	0.000		47.90 ± 6.82	90.10 ± 4.13	-23.29	0.000
*t* value	-0.548	-0.297				-0.000	0.036		
*P* value	0.590	0.770				1.000	0.972		

## Discussion

The ACL and posterior cruciate ligament (PCL) are critical to the stability and function of the knee, particularly under the strain of vigorous movements [1]. ACL injuries, primarily sports-related, occur most frequently among young, active individuals [1] and can lead to knee instability and a limited range of motion due to abnormal stresses and additional structural damage within the joint. These injuries may result in enduring physical and psychological effects [9, 10]. Arthroscopic ACL reconstruction has emerged as the primary therapeutic intervention for such injuries [11].

With an increase in ACL reconstruction, there has also been a rise in revision surgeries following initial procedures. Studies have revealed a retear rate of 7% after ACL reconstruction [12]. Factors such as young age and the resumption of high-intensity activities are closely linked with secondary ACL injuries [13]. Notably, young athletes who return to sports following ACL reconstruction are at a significantly elevated risk of 30 to 40 times greater secondary injuries compared to their uninjured peers [14]. Among individuals under 25, the retear rate is reported at 10% [15]. Long-term follow-ups over a decade indicate a graft tear rate of 6.2%, with about 10.3% of cases resulting in clinical failure [16]. The highest incidence of retears has been observed in young males, with 18% occurring approximately 1.8 years post-surgery [17]. The efficacy of ACL reconstructions is contingent upon several factors, including patient selection, surgical techniques, and postoperative rehabilitation. A critical element of the surgical approach is the precise creation of the bone tunnel, particularly the placement of the femoral bone tunnel, which is vital for the success of the reconstruction [18, 19].

Current ACL reconstruction techniques are divided into non-anatomic (isometric) and anatomic (non-isometric) approaches. The isometric method, which deviates from the natural anatomical positioning of the knee, can alter biomechanics and often results in less than optimal recovery of the knee joint function. In contrast, anatomical reconstruction restores the knee’s rotational stability and has demonstrated superior clinical outcomes compared to the isometric approach [20]. Van Eck et al. identified graft extension as the primary pattern of rupture in patients undergoing revision surgery following single-bundle ACL reconstruction [21]. The ACL is characterized by non-isometric behaviour across various angles of knee motion, incorporating both direct and indirect fibers at the femoral insertion. These direct fibers are essential for knee movement and fundamentally support the stability of the anterior cruciate. Therefore, the anatomical placement of the ACL’s femoral tunnel should target these direct fibers [22]. Single-bundle anatomical reconstruction requires that the restored ligaments be precisely positioned in the anterior and medial bundles to ensure minimal tension and optimal isometry. Andrew et al. delineated the IDEAL criteria for optimal femoral tunnel positioning in ACL reconstruction, comprising Isometric positioning, Direct fiber entry point alignment, the center of the Anterior medial bundle, an Internal anatomical stop, and Low tension maintenance (IDEAL) [23]. The designated IDEAL point is located near the anterior inner bundle, proximal to the isometric point, and aligns with the direct fiber entry of the ACL. An IDEAL ACL reconstruction aims to situate the graft with anatomical precision, thereby fulfilling the conditions of isometry and minimal tension during movement, thus diminishing the potential for reconstruction failure due to inconsistent graft tension in postoperative knee dynamics [5]. While the concept of IDEAL positioning has garnered consensus among surgeons, its intraoperative identification largely depends on the surgeon’s expertise, presenting significant challenges for novice surgeons.

Intraoperatively, the direct visualization of the IDEAL point poses substantial difficulties, compounded by the challenge of identifying the ACL’s stop point, especially when the femoral and tibial stop points are misaligned. The limited availability of anatomical markers during surgery complicates the achievement of precise IDEAL positioning. Jaecker et al. employed Transtibial (TT) and Anteromedial (AM) portal techniques in their initial ACL reconstructions, revealing that 77.2% of femoral tunnels and 40.1% of tibial tunnels were non-anatomically placed [24]. Intriguingly, no significant correlation was found between tunnel placements in the TT and AM techniques [24]. While femoral eccentric guides are commonly used to direct the femoral side during ACL reconstruction, they are fraught with risks of inaccuracies in needle entry positioning and occasional shifts in the registration point towards a more proximal and posterior location. Some guides are oriented so posteriorly that they jeopardize the posterior cortical thickness, reducing it to less than 5 mm. It is argued that femoral offset guides may fail to identify optimal bony attachments on the femur [25]. In this study, when employing a femoral offset guide, its placement was aligned to the cartilage boundary transition behind the lateral femoral condyle. However, variability in patients’ bony anatomy at this point sometimes led to guide misalignment, potentially resulting in fractures at the posterior edge of the lateral femoral condyle.

The clinical integration of 3D printing technology has yielded notable outcomes, spanning the creation of medical prototypes, surgical aids, and implantable materials. By leveraging patient-specific imaging data, 3D printing provides tailored solutions to unique clinical challenges. Zee, MJM et al. demonstrated that patient-specific 3D-printed surgical guides could enhance the accuracy and consistency of femoral tunnel positioning in ACL reconstructions [26]. Rankin et al. analyzed MRI data to determine the ACL femoral endpoints and noted that while 3D-printed femoral guides meet anatomical positioning standards, their large size presents significant clinical challenges [27]. Liu et al. achieved good therapeutic outcomes and reduced intraoperative positioning time by using a personalized 3D-printed navigation template for reconstructing the ACL at the ligament’s femoral endpoint center [28]. Lan et al. found that computer-assisted 3D personalized guide plate positioning methods were more effective for lateral femoral tunnel placement in knee joint ACL reconstructions, significantly reducing positioning time [29]. Wang et al. observed that 3D-printed guide plates facilitated individualized ACL reconstruction, improving the accuracy of femoral tunnel positioning, enhancing safety and efficiency, reducing surgical and positioning times without increasing incision length, and achieving higher functional scores and rotational stability of the knee joint, aligning with the principles of individualized ACL reconstruction [30].

Common technical errors in ACL reconstruction often involve suboptimal femoral tunnel placement [31], with studies indicating an average deviation of 12.5 mm from the optimal point when surgeons rely solely on anatomical landmarks [32]. Hence, we intend to improve surgical precision through 3D-printed guides, independent of the surgeon’s experience, by increasing intraoperative positioning references and reducing error rates. Challenges include the size of the femoral tunnel guide plates designed by Rankin et al., which are difficult to insert through arthroscopic portals [27]. The preoperative requirement by Liu et al. for bilateral knee joint CT and MRI scans to prepare femoral tunnel guide plates using a mirror image method from the normal knee’s ACL femoral side point, thereby increasing radiation exposure and costs [28]. The assumption of identical femoral endpoints in both knees may not hold in all individuals, presenting discrepancies with personalized precision treatment. Our study determined the IDEAL point for ACL femoral tunnel reconstruction preoperatively, serving as the internal entry point for creating the femoral tunnel guide device. Our guide plates and attached guide devices, designed around the medial wall of the lateral femoral condyle and the IDEAL point, have a maximum diameter of 15 mm, allowing passage through the arthroscopic surgical incision without the need for the extension.

In this research, CT data of the knee joint was collected, and Mimics image processing software was utilized for the three-dimensional reconstruction of the patient’s knee joint. Preoperatively, the IDEAL point for the ACL on the femur was identified using the quadrant technique. A bespoke femoral tunnel positioning guide was conceptualized, considering the three-dimensional anatomy of the bone and cartilage margins of the lateral femoral condyle. The production of a 3D-printed, individualized femoral tunnel positioning guide alongside a femorotibial model for ACL reconstruction followed this procedure. This approach enhances preoperative understanding of the knee joint’s anatomy, facilitates meticulous surgical planning, and utilizes patient-specific instrumentation, thereby augmenting surgical efficiency and emphasizing precision and personalization during the procedure.

This study undertook a retrospective analysis of 40 patients diagnosed with ACL ruptures. By leveraging 3D printing technology, an optimally positioned guide plate for the femoral side was designed preoperatively to aid in tailored ACL reconstruction. During surgery, fibrous tissue at the ACL femoral endpoint was removed to expose the bony anatomical foundation. The approach involved an anteromedial upper portal for observation, an anteromedial lower portal for installing the hollow guide, and an anterolateral portal for attaching the guide plate, facilitating personalized ACL reconstruction. A comparative analysis of postoperative CT data demonstrated no significant differences in the femoral bone tunnel’s position (anteroposterior dimensions), according to the proportions defined by the Quadrant method. Using a 3D-printed guide plate in ACL reconstruction has shown considerable reliability and reproducibility. This technique proves particularly beneficial for the precise preparation of the femoral aspect of the ACL, thereby enhancing localization accuracy and the precision of individualized ACL reconstruction, especially for less experienced surgeons. Twelve-month postoperative follow-ups comparing IKDC and Lysholm scores revealed no significant clinical differences between the groups. Our results are consistent with those of Liu et al., Lan et al., and Wang et al., all showing favorable outcomes [28–30]. However, this study did not account for differences in surgical time between the two methods.

Addressing the intricacies and methodologies in the design of 3D printing, the IDEAL points were identified preoperatively, with postoperative imaging used to assess the location of the bone tunnel. The Quadrant method, introduced by Bernard and widely acknowledged, employs standard lateral X-rays to measure the bone tunnel’s depth and height relative to the lateral femoral condyle’s anterior-posterior length, height, and Blumensaat’s line. Recent applications by Zantop et al. have incorporated the Quadrant method into 3D CT reconstructions to evaluate the dimensions of the ACL osteocanal [33]. From an anatomical perspective, Zantop et al. reported the central depth of the anterior medial band to be 18.5%, with a height of 22.3%, while the posterior lateral band showed a central depth and height of 29.3% and 53.6%, respectively. Utilizing 3D CT data, Bird et al. determined the femoral stop center of the ACL to have a depth and height of 28% and 35%, respectively [34]. Moreover, values reported by de Abreu-e-Silva were 30.9% and 30% for depth and height, respectively [35]. In our facility, the femoral bone tunnel depth associated with the anterior cruciate ligament approximates 25% and 29%, aligning with the literature. For this study, the bone tunnel design in the 3D printing positioning group had a depth of 25% and 29%, with actual depths and heights being (25.39 ± 2.98)% and (28.89 ± 2.50)%, respectively. These measurements correspond with the preoperative designs, and no significant statistical differences were observed between the groups. This finding confirms that the 3D-printed guide plate adheres to preoperative specifications, offering high accuracy and reproducibility in ACL reconstruction.

## Conclusion

The findings of this study suggest that 3D printing-assisted arthroscopic IDEAL point femoral tunnel positioning and conventional arthroscopic positioning are feasible and effective for ACL reconstruction. In addition, preoperative 3D modeling using plain CT scans, in conjunction with the preparation of an IDEAL femoral side guide plate, can effectively pinpoint the optimal femoral tunnel intraoperatively. This method boasts high reliability and repeatability in preparing the femoral bone tunnel and facilitates personalized tunnel preparation. Future research may investigate the creation of elliptical, elongated, or other atypical bone tunnels.

## Data Availability

All data generated or analyzed during the present study are included in this published article.

## Abbreviations

ACL: Anterior cruciate ligament
AM: Anteromedial
CT: Computed tomography
DICOM: Digital Imaging and Communications in Medicine
IKDC: International Knee Documentation Committee Subjective Knee Form
PCL: Posterior cruciate ligament
STL: Standard Template Library
SD: Standard deviation
TT: Transtibial
